# The effect of hypoxia on chondrogenesis of equine synovial membrane-derived and bone marrow-derived mesenchymal stem cells

**DOI:** 10.1186/s12917-019-1954-1

**Published:** 2019-06-14

**Authors:** Alexis L. Gale, Renata M. Mammone, Michael E. Dodson, Renata L. Linardi, Kyla F. Ortved

**Affiliations:** Department of Clinical Studies, New Bolton Center, School of Veterinary Medicine, University of Pennsylvania, Kennett Square, PA USA

**Keywords:** Mesenchymal stem cell, Equine, Synovial membrane, Bone marrow, Normoxia, Hypoxia, Chondrogenesis

## Abstract

**Background:**

Joint injury is extremely common in equine athletes and post-traumatic osteoarthritis (PTOA), a progressive and debilitating disease, is estimated to affect 60% of horses in the USA. The limited potential for intrinsic healing of articular cartilage has prompted intense efforts to identify a cell-based repair strategy to prevent progression of PTOA. Mesenchymal stem cells (MSCs) have the potential to become an ideal source for cell-based treatment of cartilage lesions; however, full chondrogenic differentiation remains elusive. Due to the relatively low oxygen tension in articular cartilage, hypoxia has been proposed as a method of increasing MSC chondrogenesis. The objective of this study was to investigate the effect of hypoxic culture conditions on chondrogenesis in equine synovial membrane-derived MSCs (SM-MSCs) and bone marrow-derived MSCs (BM-MSCs). MSCs were isolated from synovial membrane and bone marrow collected from 5 horses. Flow cytometric analysis was used to assess cell surface marker expression including CD29, CD44, CD90, CD105, CD45, CD-79α, MHCI and MHCII. MSC pellets were cultured in normoxic (21% O_2_) or in hypoxic (5% O_2_) culture conditions for 28 days. Following the culture period, chondrogenesis was assessed by histology, biochemical analyses and gene expression of chondrogenic-related genes including *ACAN*, *COL2b*, *SOX9*, and *COL10A1*.

**Results:**

Both cell types expressed markers consistent with stemness including CD29, CD44, CD90, CD105, and MHCI and were negative for exclusion markers (CD45, CD79α, and MHCII). Although the majority of outcome variables of chondrogenic differentiation were not significantly different between cell types or culture conditions, *COL10A1* expression, a marker of chondrocyte hypertrophy, was lowest in hypoxic SM-MSCs and was significantly lower in hypoxic SM-MSCs compared to hypoxic BM-MSCs.

**Conclusions:**

Hypoxic culture conditions do not appear to increase chondrogenesis of equine SM-MSCs or BM-MSCs; however, hypoxia may downregulate the hypertrophic marker *COL10A1* in SM-MSCs.

## Background

Joint injury and cartilage damage are extremely common in equine athletes and often precipitate post-traumatic osteoarthritis (PTOA), a progressive and debilitating disease. Due to the poor intrinsic healing capabilities of cartilage, focal chondral defects are repaired with biomechanically inferior fibrocartilage following injury [1, 2]. In contrast to hyaline cartilage, fibrocartilage is unable to provide adequate compressive and tensile strength, thereby facilitating global joint degradation. Palliative care consists of non-steroidal anti-inflammatory drugs (NSAIDs) and corticosteroid therapy both of which have deleterious long-term side effects [3, 4]. Currently, no effective disease-modifying drugs that halt or reverse PTOA are available [5]. Repairing damaged articular cartilage following injury may prevent further joint degeneration and would be greatly beneficial.

Cell-based cartilage repair strategies have been intensely investigated; however, a suitable cell source for regeneration of hyaline cartilage remains elusive. In human orthopedic surgery, autologous chondrocyte implantation (ACI) has been the “gold-standard” for repair of large cartilaginous defects [6, 7]. Both autologous and allogeneic chondrocyte implantation have been described with some success in the horse [8, 9]. Despite improved clinical outcomes and healing, ACI has several limitations including the need for multiple surgical procedures, graft hypertrophy [10], and donor site morbidity [11]. Considering the limitations of chondrocyte implantation, an alternative cell source for resurfacing the articular surface, such as mesenchymal stem cells (MSCs), would be favorable.

MSCs are an ideal cell source as they are easily accessible, can be culture-expanded, and are multipotent with chondrogenic differentiation capabilities. The goal of MSC-based cartilage repair is for chondrogenesis of MSCs implanted into chondral defects facilitating replacement of hyaline cartilage. The vast majority of cell-based cartilage repair has been focused on bone marrow-derived MSCs (BM-MSCs); however, recently, synovial membrane-derived MSCs (SM-MSCs) have been investigated as a cell source due to demonstration of superior chondrogenesis in other species [12]. Synovium can be harvested in standing horses or during arthroscopic procedures, with SM-MSCs being isolated and expanded in the laboratory in preparation for chondrogenic differentiation.

Lack of complete MSC chondrogenic differentiation and progression towards the hypertrophic phenotype, with increased expression of collagen type X (*COL10A1*) remains a challenge in cell-based cartilage repair [13, 14]. Differences in culture conditions appear to be an important factor in the effectiveness of MSC chondrogenesis. Chondrocytes reside in a relatively hypoxic environment of 1–5% O_2_ (8-40mmHg) compared to other tissues in the body, including bone marrow which is at 7% O_2_ (50mmHg) [15, 16]. Traditional CO_2_ incubators are maintained at 21% O_2_ and 5% CO_2_, which may limit chondrogenesis in MSC cultures. Relative hypoxia has had variable effects on chondrogenesis thus far, with some studies demonstrating enhanced chondrogenesis in BM-MSCs and articular cartilage progenitor (ACP) pellet cultures with upregulation of collagen type II, aggrecan and SOX9 [17]. Hypoxia increases expression of hypoxia-inducible factors (HIFs), which play a significant role in signaling pathways of chondrogenesis, including SOX9, a key transcription factor of chondrogenesis [18, 19]. Ranera et al. (2013) demonstrated improved chondrogenesis in equine BM-MSCs cultured in hypoxic conditions [20]; however, studies investigating the effect of hypoxia on SM-MSCs are lacking.

Currently, there are no published studies evaluating the effects of hypoxia on equine SM-MSCs.

The main objective of this study was to compare the chondrogenic capabilities of equine SM-MSCs and BM-MSCs in hypoxic and normoxic culture conditions. We hypothesized that hypoxic culture conditions would increase chondrogenesis in both BM-MSCs and SM-MSCs but that SM-MSCs would have superior chondrogenesis compared to BM-MSCs.

## Results

### Immunophenotyping

**Fig. 1:**
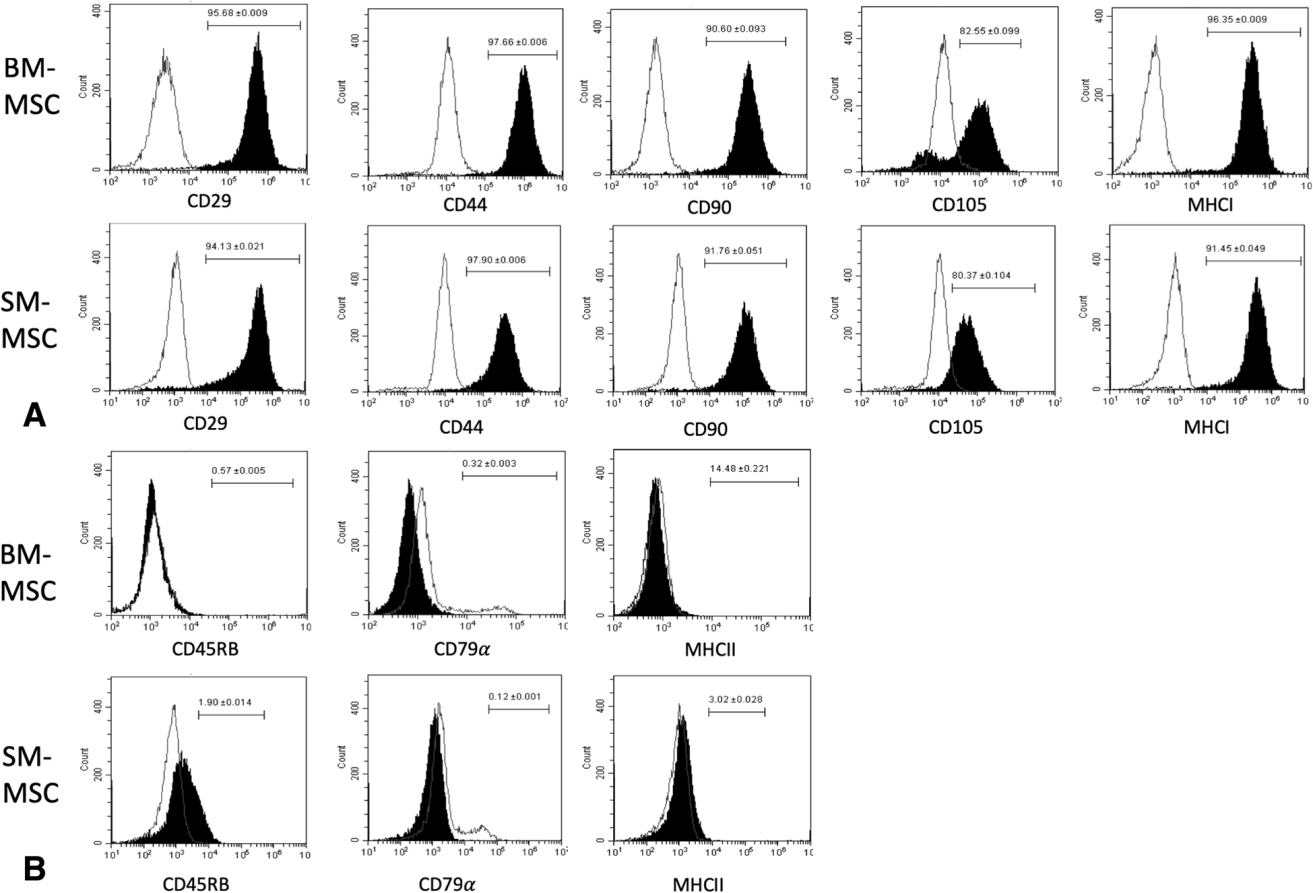
Characterization of BM-MSCs and SM-MSCs using flow cytometric quantification of cell surface marker expression. **a** Expression of cell surface markers expected to be positive in MSC populations and **b** expression of cell surface markers expected to be negative in MSC populations. The white histograms represent isotype controls and black histograms represent respective cell surface marker staining. The mean ± SEM percentage of positive cells is in the corner of each histogram. Each histogram is a representative result of 5 horses

The immunophenotypes of passage 2 (P2) BM-MSCs and SM-MSCs, as analyzed by flow cytometry, were similar between cell types with both cell types displaying cell surface antigen expression characteristic of MSCs (Fig. 1). BM-MSCs and SM-MSCs were strongly positive for expression of CD29, CD44, CD90, CD105 and MHCI. BM-MSCs and SM-MSCs were also negative for expression of the hematopoietic cell surface markers CD45RB and CD79α. As demonstrated in previous studies in our laboratory (unpublished data), BM-MSCs had variable expression of the exclusion marker, MHCII, with 14.48 ± 0.221% cells expressing MHCII, while expression of MHCII by SM-MSCs was negligible 3.02 ± 0.028%.

### Chondrogenic differentiation

**Fig. 2:**
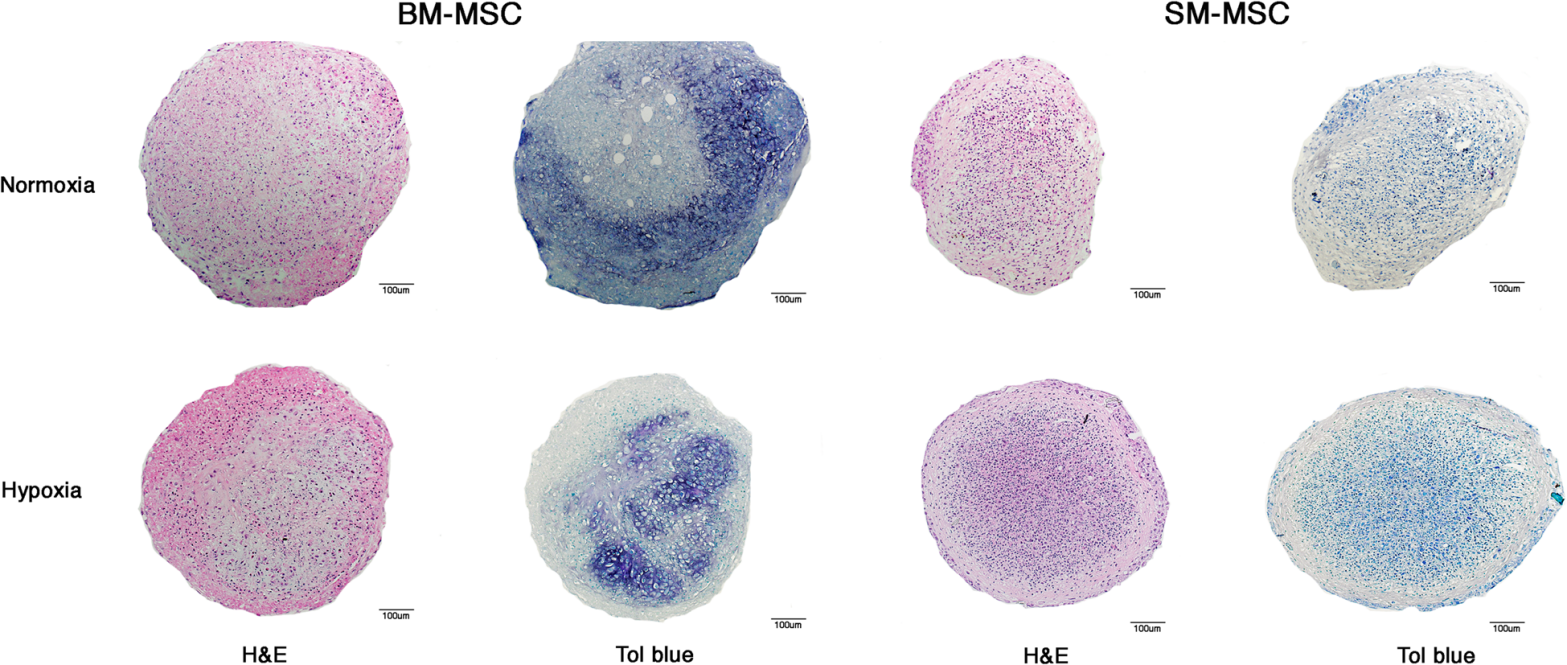
Photomicrographs of BM-MSC and SM-MSC pellets cultured in normoxic (21% O_2_) and hypoxic (5% O_2_) conditions for 28 days. Pellets were stained with H&E and toluidine blue (scale bar = 100 μm)

Chondrogenic differentiation potential of pellet cultures of SM-MSCs and BM-MSCs in normoxic and hypoxic conditions was compared by assessing MSC pellets at the end of a 28-day period. Grossly, normoxic BM-MSC pellets were larger and rounder than normoxic SM-MSC pellets, while hypoxic BM-MSC and SM-MSC pellets were similar in size and shape to normoxic BM-MSC pellets (Fig. 2). Histologically, BM-MSC pellets cultured under normoxic and hypoxic conditions exhibited more intense toluidine blue staining, consistent with proteoglycan deposition, than SM-MSC pellets cultured in either oxygen tension (Fig. 2).

**Fig. 3:**
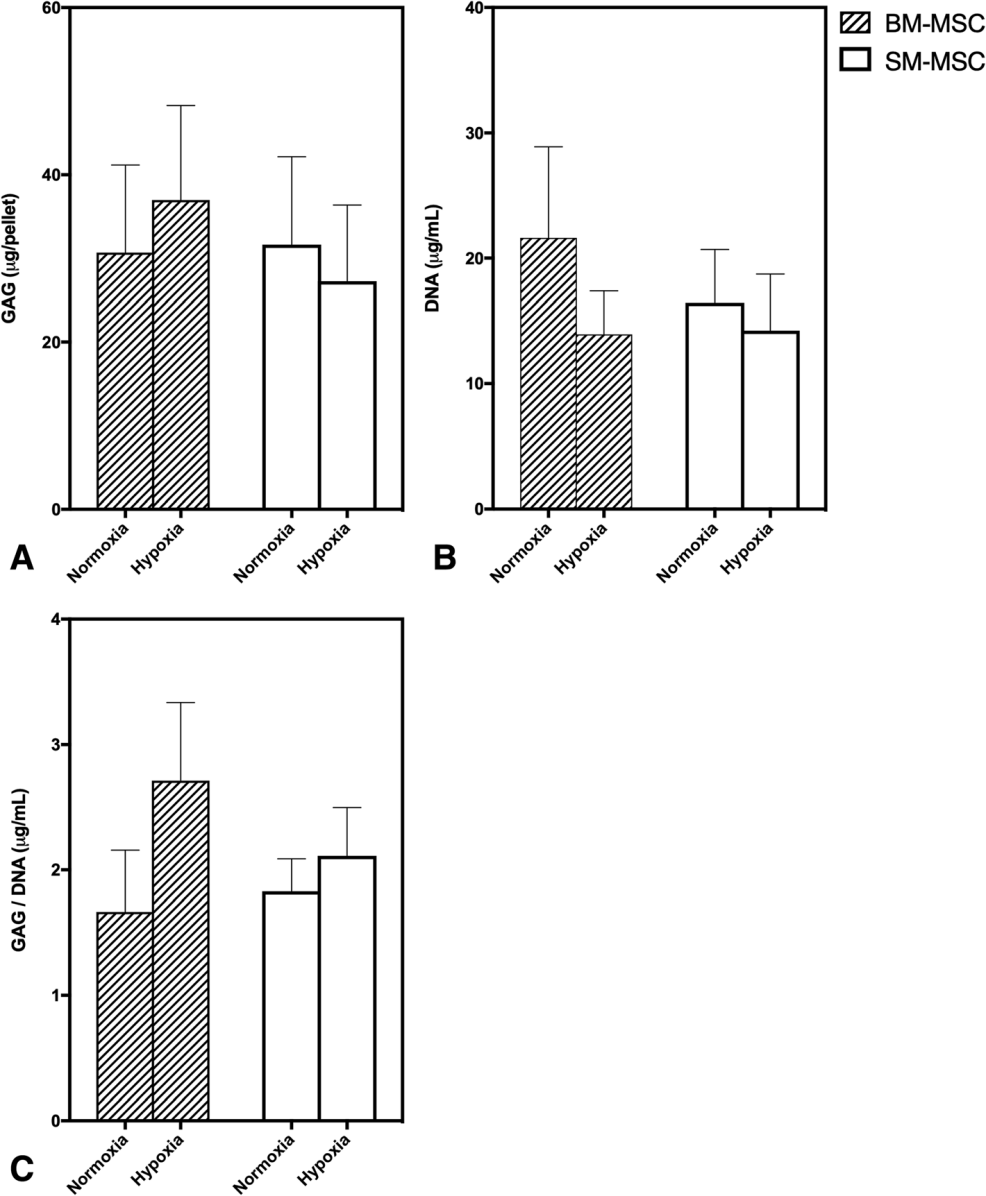
Mean ± SEM **a**) GAG, **b**) DNA, and **c**) GAG:DNA content in BM-MSC and SM-MSC pellets cultured in normoxic (21% O_2_) and hypoxic (5% O_2_) conditions for 28 days

Glycosaminoglycan content, as quantified by the DMMB assay, was not significantly different between any of the treatment groups (Fig. 3). Additionally, DNA content and GAG/DNA ratio was not significantly different between any of the treatment groups (Fig. 3).

**Fig. 4:**
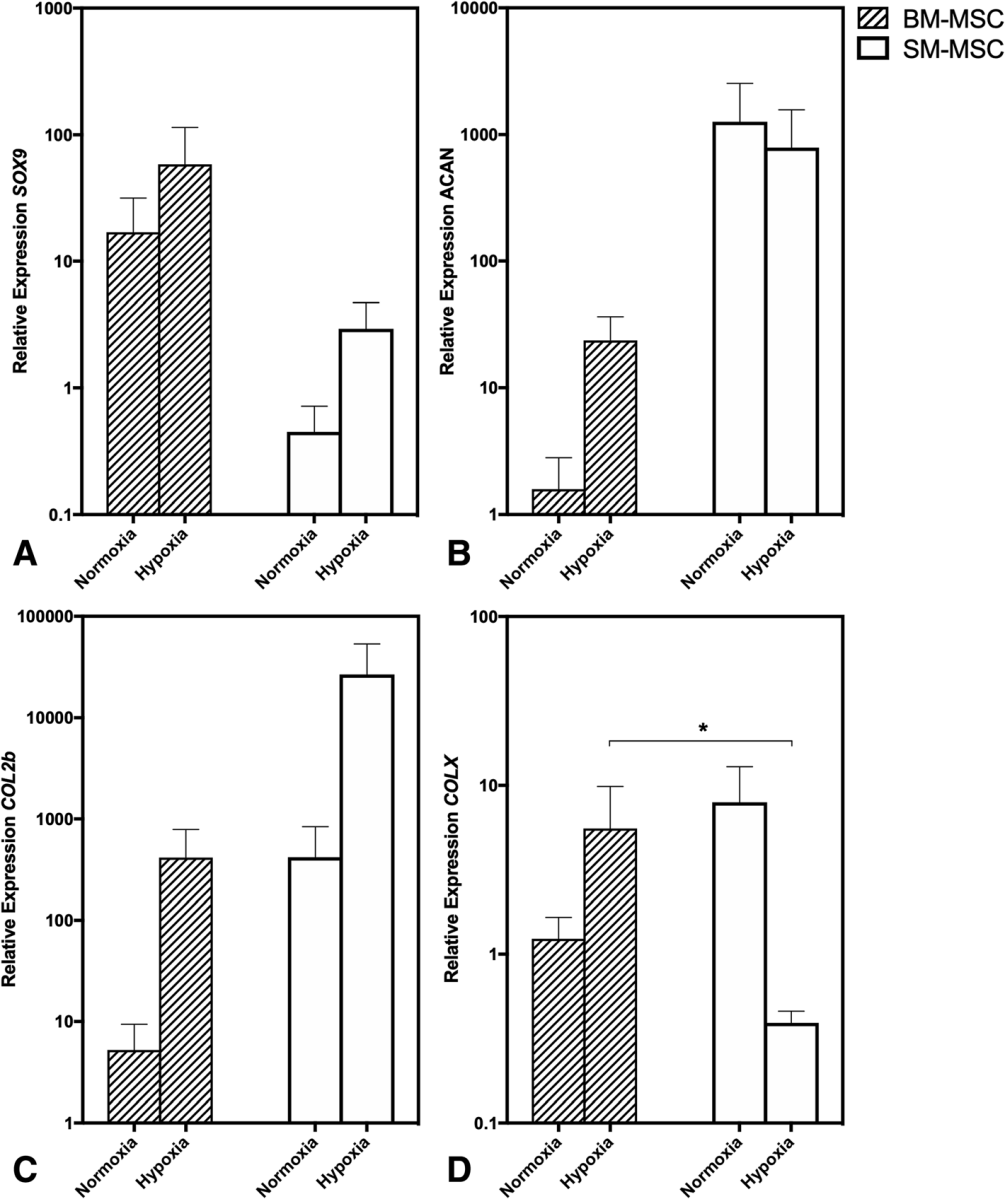
Relative expression (mean ± SEM) of chondrogenic-related genes including *SOX9*, *ACAN*, *COL2b* and *COL10A1* for BM-MSC and SM-MSC pellets cultured in normoxic (21% O_2_) and hypoxic (5% O_2_) conditions for 28 days. Expression is relative to normoxic BM-MSC pellets. *18S* was used as a reference gene in all analyses. * *p* < 0.05

Expression of markers of chondrogenesis including *SOX9*, *ACAN*, and *COL2b* displayed variability as noted in Fig. 4. Expression of *SOX9* was higher in normoxic and hypoxic BM-MSCs compared to SM-MSCs, whereas expression of *ACAN* and *COL2b* were both higher in normoxic and hypoxic SM-MSCs compared to BM-MSCs. There was a statistical trend for *COL2b* expression to be higher in hypoxic SM-MSCs compared to normoxic BM-MSCs (*p* = 0.0641). *COL10A1* expression, a marker of hypertrophy, was significantly lower in hypoxic SM-MSCs compared to hypoxic BM-MSCs (*p* = 0.0339) (Fig. 4).

### Adipogenic and osteogenic differentiation potential

**Fig. 5:**
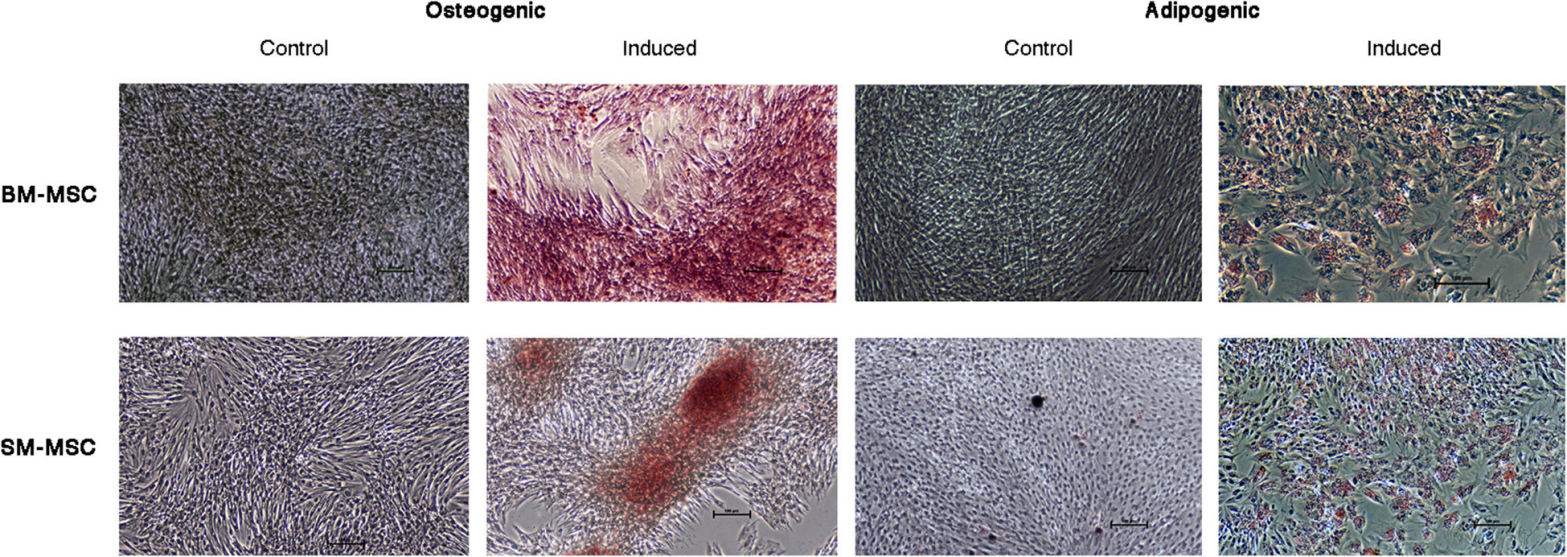
Photomicrographs of control and induced BM-MSCs and SM-MSCs stained with alizarin red (osteogenesis) and Oil Red O (adipogenesis). Osteogenically induced MSCs stained with alizarin red are positive for extracellular calcium consistent with osteogenesis. Adipogenically induced MSCs demonstrated positive Oil Red O staining of lipid droplets consistent with adipogenesis. (Bar = 100 μm)

Adipogenic differentiation was observed in both SM-MSCs and BM-MSCs with cells demonstrating lipid droplet deposition via positive staining with Oil Red O 14 days following adipogenic induction **(**Fig. 5**)**. Control SM-MSCs and BM-MSCs that were not cultured in adipogenic media did not show evidence of adipogenic differentiation histologically. Osteogenic differentiation of SM-MSCs and BM-MSCs was evident following 14 days of culture in osteogenic media. Alizarin red staining was used to assess presence of calcium, with both cell types demonstrating positive staining compared to control cells **(**Fig. 5**)**. Control cultures of SM-MSCs and BM-MSCs cultured in basal medium did not show any histologic evidence of differentiation.

## Discussion

The aim of this study was to investigate the effects of hypoxia on chondrogenesis of equine SM-MSCs and BM-MSCs. Although many studies have evaluated chondrogenesis of equine MSCs derived from different sources, including bone marrow, synovial fluid, and adipose tissue, consistent and complete chondrogenic differentiation remains elusive [21–23]. In attempts to improve chondrogenesis, different culture conditions have been investigated, including relative hypoxia as chondrocytes reside in a low oxygen environment in the body(~ 1–5% O_2_), especially compared to standard incubator oxygen tension (~ 21% O_2_) [16, 24, 25]. To date, no studies have evaluated the effects of hypoxia on equine SM-MSCs. In this study, we demonstrated that overall chondrogenic differentiation was not significantly different between SM-MSCs and BM-MSCs cultured in either normoxic or hypoxic conditions. However, we found that *COL10A1* expression, a marker of chondrocyte hypertrophy, was significantly downregulated in SM-MSCs cultured in hypoxia.

In order to evaluate our cell populations for cell surface markers consistent with MSCs prior to chondrogenesis in hypoxic conditions, we evaluated the immunophenotypes of SM-MSCs and BM-MSCs using flow cytometry. We found that both populations of cells were positive for cell surface markers consistent with MSCs including CD29, CD44, CD90, and CD105 and negative for exclusion markers including CD45, CD79α, and MHCII. As demonstrated in previous studies in our laboratory (unpublished data), expression of MHCII was negligible in SM-MSCs (mean 3.02%), while interestingly BM-MSCs had more variable expression of MHCII (mean 14.48%) with 47.24% of cells expressing MHCII in one horse. MHCII is generally considered an exclusion marker; however, variability of MHCII expression in equine BM-MSCs has been shown repeatedly by our laboratory (unpublished data) and others [26]. These findings suggest that equine BM-MSCs may exhibit more variability in MHCII expression than equine SM-MSCs, which may be important when considering the clinical application of allogeneic MSCs. Increased immunogenicity and cell rejection has been associated with increased expression of MHCII by equine BM-MSCs and has been associated with increased immunogenicity due to allorecognition [26]. In this study we did not investigate the effect of hypoxia on immunophenotype or cell viability as hypoxia has been previously shown to have no significant effect on either factor in equine MSCs [27].

Collagen type X expression is often increased in osteoarthritis and is used as a marker of the undesirable hypertrophic phenotype in chondrocytes and chondrogenically differentiating MSCs. The majority of studies have demonstrated that hypoxia is effective at downregulating *COL10A1* expression and protein synthesis [28–30], although upregulation has been less frequently reported [31, 32]. In the study reported here, we found that *COL10A1* expression was lowest in SM-MSCs cultured in hypoxia and this was significantly downregulated compared to BM-MSCs cultured in hypoxia. This could have important implications for future studies looking to optimize culture conditions for SM-MSCs in which chondrogenesis is desirable.

Despite decreased *COL10A1* expression in SM-MSCs, there did not appear to be a significant difference in chondrogenesis between cell types or culture conditions. In fact, only moderate chondrogenesis was apparent at the end of the 28-day culture period when differentiated pellets were evaluated histologically and biochemically. Lack of complete chondrogenesis of equine MSCs continues to be significant hurdle with many studies investigating the effects of different culture conditions including oxygen tension, cell type, and growth factors [24, 25]. Interestingly, Anderson et al.(2016) recently demonstrated that chondrogenic potential and lack of the hypertrophic response were present in low oxygen tension; however, this only held true for cells with high intrinsic chondrogenic capacity at baseline prior to differentiation [17]. Similar variability in chondrogenic capacity of cells has been demonstrated by others [33, 34], highlighting the importance of cell-to-cell variation in MSC cultures. Although different culture conditions, such as hypoxia, may slightly alter chondrogenic capacity, pre-sorting cells using fluorescent-activated cell sorting (FACS) such that cells with high intrinsic chondrogenic potential are selected may be a far more useful tool. For example, enhanced chondrogenesis has been previously demonstrated by selecting for LNGFR+THY-1+ [34] cells or CD105+ cells [35].

Hypoxia is thought to promote chondrogenesis through hypoxia-inducible factor pathways including HIF-2α-mediated induction of *SOX-9* [36] and HIF-1α-mediated inhibition of *COL1A1* [37]. In this study, there was increased expression of *SOX-9* and *COL2b* in BM-MSCs and SM-MSCs cultured in hypoxia compared to normoxia although these increases did not reach statistical significance. This may be in part due to the intra- and inter-animal variability of MSC populations. Additionally, culturing MSCs in pellet form may represent an inherent issue with oxygen tension as there is a natural gradient created across the pellet, despite constant incubator conditions, leading to cells within the pellet being exposed to variable oxygen tensions. For example, Markway et al. (2010) found that micropellets (~ 170 cells/micropellet) demonstrated superior chondrogenesis in hypoxic conditions compared to larger pellets (~ 2 × 10^5^ cells/pellet) [38].

Another important factor involved in chondrogenesis appears to be pre-differentiation MSC expansion conditions. Ranera et al. (2013) compared the effect of MSC expansion conditions on future chondrogenesis in normoxic conditions and found that cells expanded in hypoxic conditions had increased ECM formation during 21-day pellet culture when compared to pellets formed from cells expanded in normoxic conditions. Pellets formed from hypoxia-expanded cells demonstrated increased GAG content and more intense Alcian blue and Safranin O staining [20]. Similar results have been shown in human BM-MSCs [39] and ovine BM-MSCs [40], with cells that were expanded in hypoxic conditions prior to pellet culture displaying more robust chondrogenesis regardless of culture conditions (normoxia or hypoxia) during pellet culture.

In the study reported here, MSCs were expanded under normoxic conditions prior to pellet formation, however, hypoxic expansion of cells may be indicated in future studies.

Few studies have investigated the effect of oxygen tension on chondrogenesis of SM-MSCs or synovial fluid-derived MSCs (SF-MSCs). As described for other cell types, the effect of hypoxia on chondrogenesis of these cell types appears to be variable. Both Bae et al. (2018) and Li et al. (2011) showed improved chondrogenesis in human SM-MSCs cultured under hypoxic conditions [41, 42]. However, Ohara et al. (2016) found no effect of hypoxia on the chondrogenic potential of human SM-MSCs and Neybecker et al. (2018) found minimal effects of hypoxia on chondrogenesis of human SF-MSCs obtained from OA joints [43, 44]. To the authors’ knowledge, this is the first study describing the effect of hypoxia on chondrogenesis of equine SM-MSCs. Similar to other studies, we did not detect a major effect of hypoxia on chondrogenesis of SM-MSCs.

Overall, considerable variability appears to exist within and between equine MSC populations. Although hypoxia may inhibit *COL10A1* expression in SM-MSCs, further refinement of culture conditions including pre-sorting and selection of cells with high chondrogenic potential and MSC expansion in hypoxic conditions should be considered to optimize chondrogenesis. Additionally, culturing MSCs in three-dimensional scaffolds could be considered as this has shown to improve chondrogenesis in previous studies [45].

## Conclusions

Enhanced cartilage repair using chondrogenically differentiated MSCs would be an ideal clinical resource; however, chondrogenesis of equine MSC cultures continues to represent a significant challenge. Synovial membrane-derived MSCs did not demonstrate improved chondrogenesis under hypoxic conditions. Further optimization of culture conditions is indicated for equine MSCs with efforts focused on pre-selection of MSCs with superior chondrogenic differentiation capabilities using FACS and expansion of MSCs in hypoxic conditions prior to induction of chondrogenesis.

## Methods

### Animals

Five systemically healthy adult horses (2–7 years) being euthanized at the University of Pennsylvania for reasons unrelated to the study were used to tissue collection. The study was performed following approval by the University of Pennsylvania Institutional Animal Care and Use Committee (IACUC #805973).

### MSC isolation and culture

Bone marrow was collected under sterile conditions from the sternebrae of horses immediately following euthanasia. Using an 11-gauge Jamshidi bone marrow biopsy needle (VWR Scientific, Bridgeport, NJ) containing 10,000 U of heparin, 40 mL of bone marrow was aspirated. Bone marrow samples were processed via density centrifugation with Ficoll-Paque Plus (GE Healthcare, Chicago, IL, USA) prior to seeding into flasks containing medium consisting of Dulbecco’s Modified Eagle Medium (DMEM) with 1 g/L of D-glucose, 2 mM L-glutamine, and 1 mM sodium pyruvate (ThermoFisher Scientific, Hampton, NH), penicillin (100 U/mL)-streptomycin (100 μg/mL) solution (Invitrogen, Carlsbad, CA), 10% fetal bovine serum (FBS) (VWR Life Science Seradigm, VWR, Radnor, PA), and basic fibroblastic growth factor (bFGF, 1 ng/mL) (Invitrogen, Carlsbad, CA). Media was changed every 48 h.

Synovial membrane was collected from the same horses immediately following bone marrow aspiration. All synovial membrane was collected aseptically from the dorsal aspect of the antebrachiocarpal and middle carpal joint of grossly normal carpi. Following harvest, synovial membrane was rinsed in phosphate buffered saline (PBS) with penicillin (100 U/mL) and streptomycin (100 μg/mL). Synovial membrane (~ 400 mg) was then finely cut into small pieces with a #10 scalpel blade and incubated at 37 °C in 200 μL FBS for 20 min, as previously described [46]. Samples were re-suspended and cultured in DMEM with 4.5 g/L D-glucose, 2 mM L-Glutamine, and 1 mM sodium pyruvate, penicillin (100 U/mL)-streptomycin (100 μg/mL) solution, and 10% FBS. Media was changed every 48 h. Synovial membrane pieces were maintained in the flask until migration of MSCs was confirmed by the presence of MSC colonies attached to the tissue culture flasks.

Both BM-MSCs and SM-MSCs were passaged when they reached ~ 80% confluency using Trypsin-EDTA Cell Dissociation Reagent (ThermoFisher Scientific, Waltham, MA). Passage 2 (P2) cells used for all differentiation assays. Cell number and viability was determined using the Cellometer Auto 2000 Cell Viability Counter (Nexcelom Bioscience, Lawrence, MA) and ViaStain™ AOPI staining solution (Nexcelom Bioscience LLC, Lawrence, MA).

### Immunophenotyping of MSCs

Passage 2 BM-MSCs and SM-MSCs were immunophenotyped using flow cytometry. Following trypsinization, cells (6 × 10^4^) were placed in 96-well round bottom plates and washed twice with PBS. Cell pellets were resuspended in 100 μL of PBS with 0.5% bovine serum albumin (BSA) (Sigma Aldrich, St. Louis, MO) and 0.02% sodium azide (ThermoFisher scientific, Waltham, MA) and incubated at 4 °C for 20 min. Cells were then incubated with 50 μL of the appropriate primary antibody at 4 °C for 45 min, rinsed twice with PBS, and then resuspended in the secondary antibody (50 μL) when appropriate and incubated at 4 °C for 45 min. After the final PBS rinse, the pellets were re-suspended in 200 μL of PBS containing 7-AAD (7-Aminoactinomycin D, ThermoFisher scientific, Waltham, MA) as a viability stain. Cells were stained with anti-CD29, CD44, CD90, CD105, CD45, CD-79α, MHCI and MHCII antibodies and isotype controls were used to establish fluorescent gates **(**Table 1**)**. Flow cytometry and subsequent analysis were performed using the Cytoflex S Benchtop Flow Cytometer and CytExpert Software, version 1.0 (Beckman Coulter Inc., Brea, CA).

**Table 1:** Antibodies used for flow cytometric analysis of equine cell surface markers

Antibody	Clone/ Isotype	Host Species	Target Species	Fluorophore	2^o^ Antibody	Company	Dilution for 1^o^ Antibody
CD29	TMD29/IgG1	Mouse	Human	APC	Yes Goat anti-mouse IgG	EMD Millipore	1:100
CD44	IM7/IgG2b	Rat	Human	FITC	No	Thermo IM7	1:80
CD90	?/IgM	Mouse	Canine, Equine	RPE	No	WSU Monoclonal Antibody Center	1:200
CD105	SN6/IgG1	Mouse	Human	Alexa 488	No	Bio Rad	1:10
CD45RB	?/IgM	Mouse	Equine	RPE	No	WSU Monoclonal Antibody Center	1:200
CD79α	HM57/IgG1	Mouse	Human	Alexa 647	No	Bio Rad	1:200
MHCI	cz3/IgG2b	Mouse	Equine	APC	Yes Goat anti-mouse IgG	Gift^a^	1:100
MHCII	cz11/IgG1	Mouse	Equine	APC	Yes Goat anti-mouse IgG	Gift^a^	1:200
Isotype Control	Corresponding MAB		Target Species	Fluorophore		Company	Dilution
IgG1	To CD29		Mouse	APC		Abcam	1:100
IgG2b	To CD44		Rat	Alexa 488		Abcam	1:100
IgM	To CD90		Mouse	PE		Abcam	1:200
IgG1	To CD105		Mouse	Alexa 488		Abcam	1:200
IgM	To CD45RB		Mouse	PE		Abcam	1:200
IgG1	To CD79α		Mouse	Alexa 647		Abcam	1:400
IgG2b	To MHCI		Mouse	APC		Abcam	1:100
IgG1	To MHCII		Mouse	APC		Abcam	1:100

^a^Gifts from Dr. Doug Antczak, Cornell University, Ithaca, New York, USA

### Chondrogenic differentiation assay

For chondrogenic differentiation, 500,000 P2 cells were pelleted in 15 mL conical tubes via centrifugation at 400 g for 5 min. After 48 h in the appropriate basal media for the cell type, chondrogenesis was induced with chondrogenic media containing of DMEM with 4.5 g/L D-glucose with 1% sodium pyruvate and L-Glutamine (4 mM), HEPES buffer (25 mM), penicillin (100 U/mL)-streptomycin (100 μg/mL) solution supplemented with transforming growth factor-β3 (0.01 μg/mL) (ThermoFisher Scientific, Waltham, MA), dexamethasone (0.4 μg/mL), 2- phospho-L-ascorbic acid (0.05 μg/mL), proline (0.04 mg/mL) (ThermoFisher Scientific, Waltham, MA), 1% insulin-transferrin-selenium solution (ThermoFisher Scientific, Waltham, MA), and 1% FBS. Pellets were maintained in normoxic (21% O_2_) or in hypoxic (5% O_2_) culture conditions for 28 days. At the end of the 28-day culture period, pellets were fixed in a 10% formalin solution prior to paraffin embedding and sectioning. Pellet sections at the thickness of 5 μm obtained from the center of the pellet were then stained with hematoxylin and eosin (H&E) and toluidine blue.

### Adipogenic and osteogenic differentiation assays

Adipogenic and osteogenic differentiation assays were performed in normoxic (21% O_2_) only to demonstrate multipotency of both cell types. For adipogenic differentiation, cells were seeded into 6-well tissue culture plates containing basal medium at a density of 5100 cells/ cm^2^. After 48 h, the medium in the treatment wells was changed to adipogenic induction medium consisting of the basal differentiation medium outlined above supplemented with biotin (8 μg/mL) (Sigma-Aldrich, St. Louis, MO), calcium pantothenate (4 μg/mL) (Sigma-Aldrich, St. Louis, MO), insulin (5.8 μg/mL) (Sigma-Aldrich, Stl Louis, MO), dexamethasone (4 μg/mL), isobutylmethylxanthine (0.1 mg/mL) (Sigma-Aldrich, St. Louis, MO), rosiglithizone (0.0178 mg/mL) (Sigma-aldrich, St. Louis, MO), 5% rabbit serum (ThermoFisher Scientific, Waltham, MA), and 3% FBS. Medium was changed every 48 h. After 6 days in induction medium, the medium was changed to adipogenic maintenance medium using the same reagents except rosiglithisone or isobutylmethylxanthine. For each horse, control SM-MSCs and BM-MSCs were maintained in the cell-type specific basal medium for the duration of the culture. Following 14 days of culture, cells were rinsed with PBS and fixed with 10% formalin before staining with Oil Red O (Sigma-Aldrich Corp., St. Louis, MO) for confirmation of lipid droplet accumulation in the cytoplasm of cells.

For osteogenic differentiation, cells were seeded into 6-well culture plates in SM-MSC or BM-MSC medium at a seeding density of 2900 cells/cm^2^. After 48 h, osteogenic differentiation medium was added containing basal differentiation medium consisting of Advanced DMEM/F12, 1% sodium pyruvate (Gibco Life Technologies, Carlsbad, CA), 25 mM HEPES buffer, 4 mM L-glutamine (ThermoFisher Scientific, Waltham, MA), and penicillin (100 U/mL)-streptomycin (100 μg/mL) solution. The basal medium was supplemented with β-glycerophosphate (2.2 μg/mL) (Sigma Aldrich, St. Louis, MO), dexamethasone (8 μg/mL), 2-phospho-L-ascorbic acid (0.05 mg/mL) (Sigma-Aldrich, St. Louis, MO), and 10% FBS. Cells are cultured in osteogenic medium for 14 days. Media was changed every 48 h. For each horse, control SM-MSCs and BM-MSCs were maintained in basal medium appropriate to the cell type for the duration of the culture. Following 14 days of culture, cells were rinsed with PBS and fixed with 10% formalin before staining with 2% Alizarin Red (Sigma-Aldrich, St. Louis, MO) at pH 4.2 for confirmation of extra-cellular calcium matrix.

### Gene expression

For assessment of chondrogenic differentiation, two pellets were collected from each treatment group. Pellets were biopulverized in liquid nitrogen using a multiple sample stainless steel biopulverizer and hammer (BioSpec Products, Inc., Bartlesville, OK). RNA was extracted using the Qiagen RNeasy Fibrous Tissue Mini Kit (Qiagen, Germantown, MD). RNA concentration and purity were quantified with a UV microspectrophotometer (NanoDrop™ One, ThermoFisher Scientific, Waltham, MA). Complementary DNA was prepared using a High Capacity cDNA Reverse Transcription kit (ThermoFisher Scientific, Waltham, MA) and an Eppendorf master cycler (Hamburg, Germany). Real-time quantitative PCR was performed using TaqMan™ Master mix and the Applied Biosystems™ QuantStudio™ 6 Flex Real-Time PCR System (Applied Biosystem, Foster City, CA). The following genes were analyzed: aggrecan (*ACAN)*, collagen type II (*COL2b)*, SRY-box 9 (*SOX9*), and collagen type X (*COL10A1)* for chondrogenesis. Primers and probes for *ACAN*, *COL2b*, and *SOX9* were designed using NCBI Primer-BLAST and Integrated DNA Technologies (IDT) PrimerQuest Tool software and synthesized by IDT (Coralville, IA) (Table 2). Primers and probes for *COL10A1* were obtained from ThermoFisher Scientific’s proprietary equine-specific gene expression assay database (ARCE46U). All samples were run in triplicate using *18S* as a reference gene. The cycle threshold (CT) values for triplicates were averaged and data were analyzed using the ΔCt method where fold change is expressed as 2^-ΔΔCt^ using normoxic BM-MSCs as the calibrator.

**Table 2:** Equine primer and probe sequences used for gene expression analyses

Gene	Primer and probe sequences
18S, 18 small ribonucleic acid	Forward, 5′- GCCGCTAGAGGTGAAATTCT-3′
	Reverse, 5′- TCGGAACTACGACGGTATCT −3′
	Probe, 5′- AAGACGGACCAGAGCGAAAGCAT-3′
*ACAN*, aggrecan	Forward, 5′-GAGGAGATGGAGGGTGAGGT −3′
	Reverse, 5′-GATGGTGATGTCCTCCTCGC-3′
	Probe, 5′-TTCACCTGTGTAGCAGATGGCGTC-3’
*COL2b*, type II collagen	Forward, 5’-GCTACACTCAAGTCCCTCAAC-3′
	Reverse, 5′-ATCCAGTAGTCTCCGCTCTT-3′
	Probe, 5′-ACCTGAAACTCTGCCACCCTGAAT-3’
*SOX9*, SRY-box 9	Forward, 5’-CTGGAGACTGCTGAACGAGA-3′
	Reverse, 5′-GAGATGTGTGTCTGCTCCGT − 3′
	Probe, 5′-AGAAGGACCACCCGGACTACAAGTA-3’

### Biochemical analyses

BM-MSC and SM-MSC pellets were collected and stored at -20 °C in medium prior to biochemical assays. The dimethylmethylene blue (DMMB) spectrophotometric assay (Sigma-Aldrich, St. Louis, MO) was used to quantify proteoglycan content in pellets digested in 0.5 mg/mL papain (Sigma Aldrich St. Louis, MO). Chondroitin-4 sulfate (Sigma-Aldrich, St. Louis, MO) was used to establish a standard curve and the optical density determined at 525 nm [47]. Total DNA content was determined using 0.5 mg/mL papain digested pellets incubated for 24 h at 65 °C. Digested samples were then mixed bisbenzimide compound (Hoescht, Sigma-Aldrich, Burlington, MA) and DNA was quantified using a fluorometric assay with an excitation wavelength of 348 nm and an emission wavelength of 456 nm. Calf thymus DNA (Sigma-Aldrich, St. Louis, MO) was used to establish a standard curve. Proteoglycan concentration was normalized to the quantity of DNA in that sample.

### Statistical analysis

Continuous values are expressed as means ± SEM. A mixed effects model was used to analyze all continuous data including cell surface marker expression, fold change gene expression, and GAG content. Horse was considered as a random effect. Statistically significant differences between treatments were determined using a Wilcoxon rank sum test. All data were analyzed using JMP14 (SAS, Cary, NC). Significance was set at *p* < 0.05.

## Data Availability

All data generated or analyzed during this study are included in this published article.

## Abbreviations

ACAN: Aggrecan
ACI: Autologous chondrocyte implantation
BM-MSC: Bone marrow-derived mesenchymal stem cell
CD: Cluster of differentiation
COL10A1: Collagen type 10
COL2b: Collagen type 2
DMMB: Dimethylmethylene blue
FACS: Fluorescent-activated cell sorting
GAG: Glycosaminoglycan
HIF: Hypoxia-inducible factor
MHC: Major histocompatibility complex
MSC: Mesenchymal stem cell
NSAID: Non-steroidal anti-inflammatory drug
P2: Passage 2
PBS: Phosphate buffered saline
PTOA: Post-traumatic osteoarthritis
SM-MSC: Synovial membrane-derived mesenchymal stem cell
SOX9: Sex determining region Y-box 9
